# Simultaneous Detection of ^12^CH_4_, ^13^CH_4_, and Related Isotope Ratio Exploiting a Frequency-Multiplexed Mid-Infrared Quartz-Enhanced Photoacoustic Sensor

**DOI:** 10.1021/acssensors.5c02871

**Published:** 2025-12-15

**Authors:** Mariagrazia Olivieri, Arianna Elefante, Giansergio Menduni, Marilena Giglio, Hongpeng Wu, Lei Dong, Pietro Patimisco, Vincenzo Spagnolo, Angelo Sampaolo

**Affiliations:** † Polysense Lab, Dipartimento Interateneo di Fisica, 18951 University and Politecnico of Bari, Via Amendola 173, Bari 70126, Italy; ‡ Institute for Photonics and Nanotechnologies, 509739 CNR IFN, Via Amendola 173, Bari 70126, Italy; § State Key Laboratory of Quantum Optics Technologies and Devices, Institute of Laser Spectroscopy, 12441 Shanxi University, Taiyuan 030006, China; ∥ Polysense Innovations Srl, Via Amendola 173, Bari 70126, Italy

**Keywords:** methane isotopologues, isotopic composition analysis, quartz-enhanced photoacoustic spectroscopy, frequency multiplexing, simultaneous detection

## Abstract

We report the development of a dual-gas Quartz-Enhanced Photoacoustic Spectroscopy (QEPAS) sensor operating in the mid-infrared range for the simultaneous detection of ^12^CH_4_ and ^13^CH_4_. The sensor employs a frequency-modulated multiplexing scheme using two distributed-feedback quantum cascade lasers to independently excite the fundamental (f_o_) and overtone (*f*_1_) vibrational modes of a quartz tuning fork coupled with resonator tubes. The *f*_0_-demodulated signal is dedicated to monitoring ^12^CH_4_, while the *f*_1_-demodulated signal selectively quantifies ^13^CH_4_, enabling the analysis of the isotopic composition of methane samples. Calibration measurements demonstrated a linear response of the QEPAS signal to varying ^13^CH_4_ concentrations in CH_4_-based samples diluted in N_2_, with a precision of 1‰ in detecting isotopic delta ratio variations for 1% CH_4_ mixtures at 0.8 s integration time. The proposed system is suitable for real-time, high-precision isotopic methane sensing aimed at applications such as environmental monitoring, geochemical tracing, and industrial process control.

## Graphical abstract

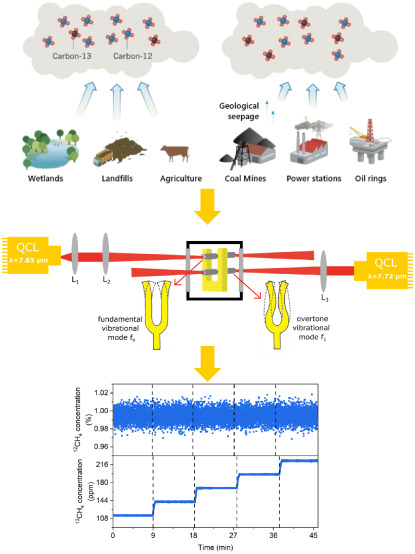

Isotope analysis has emerged as a powerful tool for emission source identification, leveraging the distinctive isotopic signatures that characterize different emissions processes. The isotopic abundance of carbon-based samples is typically expressed in terms of δ^13^C that is the deviation of the isotopic ratio ^13^C/^12^C (*R*) with respect to a standard ratio (*R*_st_) expressed in parts per thousand, i.e., $\delta^{13}C\left[‰\right]=\frac{\left(R-R_{st}\right)}{R_{st}}\times 1000$ . Monitoring the stable carbon isotopes, ^12^C and ^13^C, plays a pivotal role in many applications, such as geochemical studies, petroleum exploration, assessment of ecosystem dynamics, environmental monitoring, or medical diagnostics. It allows the origin of carbon-based emissions to be identified, biogenic,^1^ thermogenic,^2^ or pyrogenic,^3^ which are characterized by different δ^13^C ranges: [−55, −70]‰, [−25, −55]‰, and [−13, −25]‰, respectively. Methane (CH_4_) and carbon dioxide (CO_2_) are the two main anthropogenic greenhouse gases contributing to human-induced global warming. The analysis of their isotopic signature is a useful method for source apportionment and helps determining which ones contribute most to the greenhouse gas budget in urbanized areas.^4–7^ Isotopic data are widely used to investigate the biological origin of fossil fuels in geochemical studies^8,9^ as well as to understand the evolution of oil reservoirs and guide downstream operations in petroleum exploration and production.^10,11^ Natural gas hydrates are gaining enormous attention due to their characteristics of high energy density, high cleanliness, and large reserves. Currently, one of the most effective methods for detecting deep-sea natural gas hydrates involves measuring the gas dissolved in seawater, particularly by analyzing the isotopic ratios of CO_2_.^12,13^ Similarly, investigating the carbon isotopic composition of CO_2_ samples from crater fumaroles is crucial for understanding their degassing behavior and for predicting volcanic activity based on variations in δ^13^C values.^14,15^ Furthermore, stable carbon isotopes, particularly ^13^C in CO and CO_2_, are widely used in medical diagnostics through noninvasive breath tests.^16–19^ Methane is a major component of the carbon cycle in anaerobic aquatic systems, and carbon isotopic analysis provides valuable insights into the sources and sinks of CH_4_ in these environments.^20,21^ Additionally, the detection of CH_4_ and the measurement of its isotopic ratios play a key role in research about planet’s atmosphere, offering critical information about the potential presence of biological sources.^22,23^

The isotopic composition of complex carbon-based samples is typically determined using gas chromatography–mass spectrometry (GC-MS) or gas chromatography–isotope ratio mass spectrometry (GC-IRMS). These techniques provide detailed information about the mass spectra or directly measure the isotopic ratios of samples.^24–26^ However, the deployment of such systems in harsh environments, the need for sample preparations, and their bulky apparatus pose significant challenges for field applications where portable solutions are essential. Laser-based spectroscopic techniques have already demonstrated highly sensitive and selective detection of gas-phase isotopologues. Among direct absorption methods, tunable diode laser absorption spectroscopy (TDLAS) using multipass cells is one of the most representative approaches for detecting carbon isotopologues.^13,18,19,27^ In this configuration, the laser beam undergoes multiple reflections between two large diameter focusing mirrors, significantly increasing the optical path length and thereby enhancing sensitivity. However, these systems require precise optical alignment, as maintaining a stable reflection pattern within the multipass cell is critical. Cavity-enhanced absorption spectroscopy, on the other hand, utilizes a high-finesse optical cavity composed of mirrors with extremely high reflectivity.^28^ These systems are highly sensitive to misalignment, as imperfect coupling between the laser beam and the cavity modes can degrade both spectral purity and line width. Indeed, compact instruments based on cavity-enhanced techniques—such as those developed by Picarro and Los Gatos Research—are commercially available and can achieve isotopic precision better than 1‰, with integration times ranging from a few minutes to over an hour.^29,30^ Fourier Transform Infrared (FTIR) spectroscopy offers an alternative to laser-based infrared techniques by using broadband infrared radiation from a blackbody source to reconstruct the absorption spectrum of target species. The detection of the CO_2_ carbon isotopes was investigated in several works, exploiting the separation of the stretching asymmetric bands of ^12^CO_2_ and ^13^CO_2_ (∼66 cm^–1^), well within the resolving capability of standard FTIR instruments.^31,32^ For methane isotopes, characterized by a narrower spectral separation (∼10 cm^–1^), high-resolution FTIR is required, with a consequent increase in the acquisition time, which limits its suitability for real-time applications. Photoacoustic spectroscopy (PAS) is based on the detection of acoustic waves generated by the target gas molecules absorbing modulated laser light and relaxing energy via nonradiative processes, i.e., via collisions with the surrounding molecules. PAS has been demonstrated as a suitable tool for the isotope analysis of highly concentrated methane.^33^ The replacement of the acoustic cell with sharply resonant quartz tuning forks (QTF) led to the development of quartz-enhanced photoacoustic spectroscopy (QEPAS), enabling the realization of compact, highly modular gas sensors.^34–36^

A key challenge in all PAS-based methods is that sensor responsivity depends on the gas matrix: variations in background composition alter collisional relaxation dynamics of the target molecule potentially altering the sensor signal even when its concentration remains constant [31,45–47]. High-precision δ^13^C measurements therefore require careful characterization of the relaxation dynamics.

For methane-based samples, the simplest methane gas mixture that can be analyzed in nature is composed of ^13^CH_4_ and ^12^CH_4_, water vapor (H_2_O), and nitrogen (N_2_). A previous work has demonstrated that the vibration-to-translation (V–T) relaxation rates of ^12^CH_4_ and ^13^CH_4_ when colliding with N_2_ or H_2_O are effectively identical.^37^ As a result, whether ^13^CH_4_ relaxes directly through collisions with N_2_ and H_2_O or first transfers its vibrational energy to ^12^CH_4_, which then relaxes via the same collisional partners, the overall photoacoustic generation efficiency remains unchanged. This equivalence in V–T relaxation rates eliminates nonspectral cross-sensitivity in methane isotopic measurements and underlies the high reliability of QEPAS-based δ^13^C measurements. In fact, once calibrated with natural abundance samples, the sensor can also be employed to measure the isotopic ratio of mixtures with nonstandard isotopic compositions, without the need for additional compensations of the relaxation effects. A further key requirement is the ability to detect both isotopologues simultaneously in order to provide the δ^13^C information in real time, referring to the same gas sample and under the same operating conditions. These conditions become mandatory when investigating processes characterized by fast dynamics and short-term changes in the seconds time scale, since an offline sampling or a temporal delay in the detection of the two gas species can result in an unreliable assessment of the isotopic ratio. QEPAS-based multigas detection has been demonstrated in several studies, with either multiple laser sources^35,38^ or lasers with a tuning range wide enough to allow the sequential detection of different analytes.^39–41^ In such scenarios, there is a delay between detecting different gas types, corresponding to the time needed to adjust the laser wavelength to different absorption lines or to switch between multiple laser sources. As a result, these methods are termed quasi-simultaneous dual-gas detection. Simultaneous detection can be achieved connecting different acoustic detection modules in series, each coupled with a distinct laser source.^36,42^ Another method, based on a wavelength-modulation division multiplexing (WMDM) system, involves independently modulating multiple laser sources, each for a specific gas species, and combining them for detection with a single detector.^43^ The WMDM simultaneous approach has been demonstrated for the detection of CH_4_, H_2_O, and CO_2_ using a PAS sensor, with three diode lasers combined with a single spherical resonator excited at its first three radial resonance frequencies,^44^ and a QEPAS sensor, with three lasers exciting a 32 kHz QTF at its fundamental resonance frequency *f*_0_ and at a ±1 Hz shift from *f*_0_.^45^ The development of custom QTFs paved the way for utilizing the QEPAS technique in a different WMDM configuration, simultaneously exciting the fundamental and the overtone vibrational modes of the tuning fork. If the tuning fork frequency of the in-plane flexural mode is kept as low as a few kHz, the frequency of the in-plane first overtone mode, which is ∼6.2 times higher than the fundamental one, becomes accessible for QEPAS operation. Wu et al. presented a proof-of-concept for dual-gas QEPAS detection of water vapor and acetylene, utilizing a custom QTF with a fundamental and overtone mode resonance frequency equal to 2.8 and 17.78 kHz.^46^ In the research work described previously,^47^ a custom tuning fork with nearly identical resonance frequencies was used, acoustically coupled to two dual-tube acoustic resonator systems, to improve the signal-to-noise ratio of both the fundamental and first overtone QEPAS signals. Both studies demonstrated that the QEPAS signal at the QTF’s fundamental frequency is unaffected by the overtone mode vibrations and vice versa.

In this work, the WMDM configuration was implemented in a QEPAS setup for the first time to simultaneously detect the stable methane isotopologues, namely, ^13^CH_4_ and ^12^CH_4_. The fundamental and overtone modes of a QTF were excited independently and simultaneously to target ^12^CH_4_ and ^13^CH_4_ absorption lines and to perform measurements of isotopic ratio simulating the typical deviations from a CH_4_ sample in natural abundance (1.1% of ^13^CH_4_ and 98.9% of ^12^CH_4_) in the percentage range with variable concentration of the less abundant isotopologues, namely ^13^CH_4._

## Sensor Architecture

The experimental apparatus employed to achieve simultaneous detection of ^12^CH_4_ and ^13^CH_4_ is shown in Figure 1.

**Figure 1:**
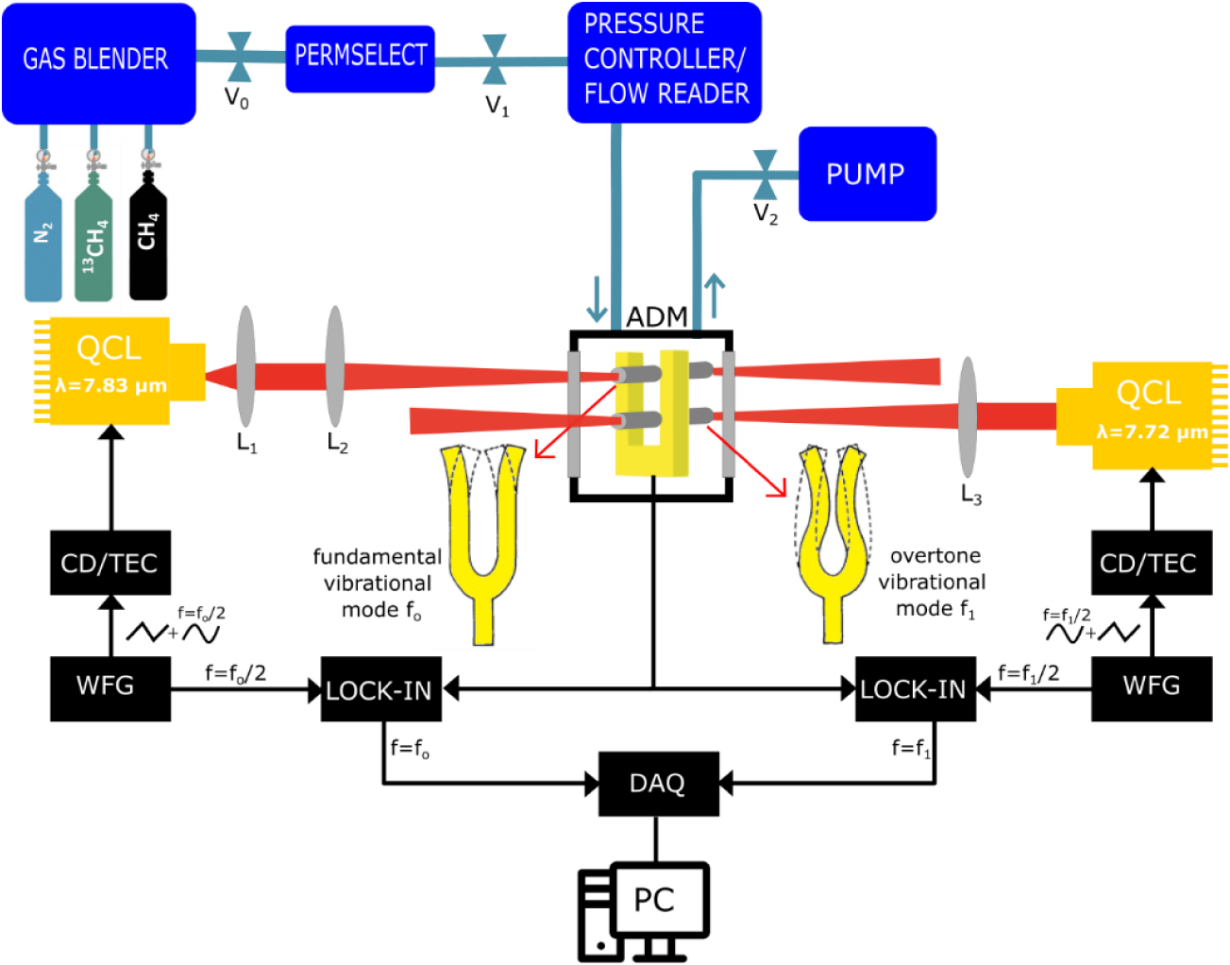
Schematic of the experimental apparatus. QCL: quantum cascade laser; ADM: acoustic detection module; CD/TEC: current driver/thermoelectric cooler; WFG: waveform generator; and DAQ: data acquisition card. A sketch of the tuning fork’s fundamental and overtone vibrational modes occurring at frequencies *f*_0_ and *f*_1_, respectively, is shown.

The dual-gas spectrophone (QTF+ tubes) is enclosed in the gas cell, forming the acoustic detection module (ADM). The gas cell is equipped with two ZnSe windows antireflection coated in the range of 7–12 μm on both the front and the back side and has external dimensions 5 × 5 × 5 cm^3^. The employed tuning fork has two rectangular prongs with a length of 17 mm, a thickness of 1 mm, a quartz crystal width of 0.25 mm, and a prong spacing of 0.7 mm.^48^ These QTF geometrical parameters lead to a fundamental frequency as low as 2.88 kHz, while the first overtone mode resonates at ∼17.78 kHz. The fundamental mode has an antinode point on top of the QTF, while the first overtone mode has two antinode points, one coincident with that of the fundamental mode and the other one close to the middle of the prong. Two pairs of acoustic resonator (AR) tubes were employed to simultaneously enhance the fundamental and the overtone vibrational modes, using the same configuration described previously.^47^ One pair of ARs tubes was located at 2 mm from the top of the prongs, i.e., near the fundamental antinode point, and the other pair at 9.5 mm from the top of the prongs, i.e., near the lower antinode point of the overtone mode. For the first overtone mode, the optimal length for QTF-AR tubes coupling falls between 4.8 and 9.6 mm, and a tube length of 8.5 mm was employed.^47^ For the fundamental mode, however, the optimal length would be between 30 and 60 mm, requiring a larger acoustic detection module, which is impractical for compact design. To address this, 9.5 mm long tubes were selected, slightly longer than those used for the overtone mode. As a result, a smaller enhancement of the QEPAS signal is expected for the fundamental mode, with respect to the first overtone one. In addition, tuning forks’ fundamental vibrational mode exhibits a lower quality factor as compared to the overtone one.^49^ Therefore, the fundamental mode was used to detect the most abundant methane isotopologue, namely, ^12^CH_4_, while the overtone mode was used for the detection of ^13^CH_4_. Two quantum cascade laser (QCL) sources at 7.82 μm (QCL1) and 7.72 μm (QCL2) were simultaneously focused within the spectrophone to excite both the fundamental and overtone vibrational modes, enabling the simultaneous detection of absorption transitions associated with ^12^CH_4_ and ^13^CH_4_, respectively. The QCL1 beam was collimated by using a molded IR aspheric lens with a focal length of 4 mm (*L*_1_) and then focused through the upper AR tubes by means of a Zn–Se lens having a focal length of 100 mm (*L*_2_) to excite the fundamental vibrational mode of the spectrophone. The QCL2 beam was focused through the lower AR tubes by means of a Zn–Se lens having a focal length of 50 mm (*L*_3_) to excite the overtone vibrational mode of the spectrophone. The QCL1 and QCL2 focused beams were measured by acquiring the laser beam profile in the focal plane with a Spiricon Pyrocam IIIHR, resulting in diameters lower than the tube diameter for both lasers. QEPAS signals were detected using the wavelength modulation technique with 2f-detection. A voltage ramp and a sinusoidal dither were applied to the laser source to finely tune the laser emission wavelength and modulate each laser at half of the corresponding excitation frequency, namely, *f*_0_ for the fundamental mode and *f*_1_ for the overtone mode. The QTF signal was converted into a voltage signal using a trans-impedance preamplifier (not shown in the figure) and then was simultaneously demodulated at the fundamental and first overtone frequencies using two lock-in amplifiers (EG&G 7265) and an integration time of 100 ms. A data acquisition card (National Instrument USB 6361) and a LabVIEW-based software were used to acquire the demodulated signal. The gas handling system was composed of an MCQ Instrument Gas Blender GB-100, used to manage the flow rate for three gas channels and produce the desired gas mixture and an MKS-type 649 pressure controller/flow meter, in combination with a needle valve and a pump used to fix the gas pressure and monitor the flow rate inside the gas line. A Nafion humidifier (PermSelect PDMSXA) was placed downstream of the gas mixer to humidify the samples, fixing the H_2_O concentration for all measurements. Relative humidity and temperature within the gas line were measured by an IST AG HYT 271 sensor positioned near the ADM (not shown in the figure). Measurements were performed using two gas cylinders, one containing a certified concentration of 0.1% ^13^CH_4_ in N_2_ and the other containing a certified concentration of 10% CH_4_ in N_2_, with an isotopic composition of 9.89% of ^12^CH_4_ and 0.11% of ^13^CH_4_. Pure N_2_ was employed as a diluent.

## Experimental Results and Discussion

### Preliminary Characterization

The preliminary characterization aims at identifying the most suitable absorption lines to be targeted for the detection of the isotopologues based on the following criteria. Mid-infrared absorption bands are typically chosen for the sensitive detection of both isotopologues because their line strength is approximately 2 orders of magnitude greater than that of near-infrared bands. Another critical factor is avoiding spectral interference from common background gases such as water vapor (H_2_O) and heavier hydrocarbons like ethane (C_2_H_6_) and propane (C_3_H_8_). Moreover, due to the mass difference of the carbon isotopes, their spectra are shifted relative to each other by approximately 10 cm^–1^.^50^ As a result, careful selection of absorption lines and operating pressure is essential to achieving complete separation of their respective 2f-QEPAS signals.

The 7.81 μm band (1200–1380 cm^–1^) offers higher selectivity for methane detection compared to the 3.32 μm band (2400–3170 cm^–1^), despite the latter featuring absorption lines with approximately twice the intensity. According to spectral data from the HITRAN and PNNL databases, the 7.81 μm range experiences significantly less spectral overlap with H_2_O, C_2_H_6_, and C_3_H_8_.^50,51^ Furthermore, reduced Doppler broadening at higher wavelengths allows for a greater separation degree of the absorption profiles associated with different gas species, especially when operating at low pressure. Based on this discussion, selective and sensitive optical detection of methane can be achieved by targeting absorption lines within the 7.81 μm band, also benefiting from the availability of high-power quantum cascade lasers (QCLs) in this spectral region. The laser sources selected for this investigation are distributed feedback quantum cascade lasers (DFB-QCLs) with center wavelengths of around 7.82 μm (QCL1) and 7.72 μm (QCL2). To evaluate their suitability for detecting methane isotopologues, simulations of the absorption cross-section and line strength were performed using the HITRAN database. The simulated gas mixture consisted of 1% methane in natural isotopic abundance (0.989% ^12^CH_4_ and 0.011% ^13^CH_4_), diluted in wet N_2_ (1% H_2_O). The obtained results are shown in Figure 2, at 75 and 760 Torr and at a room temperature of 23 °C.

**Figure 2:**
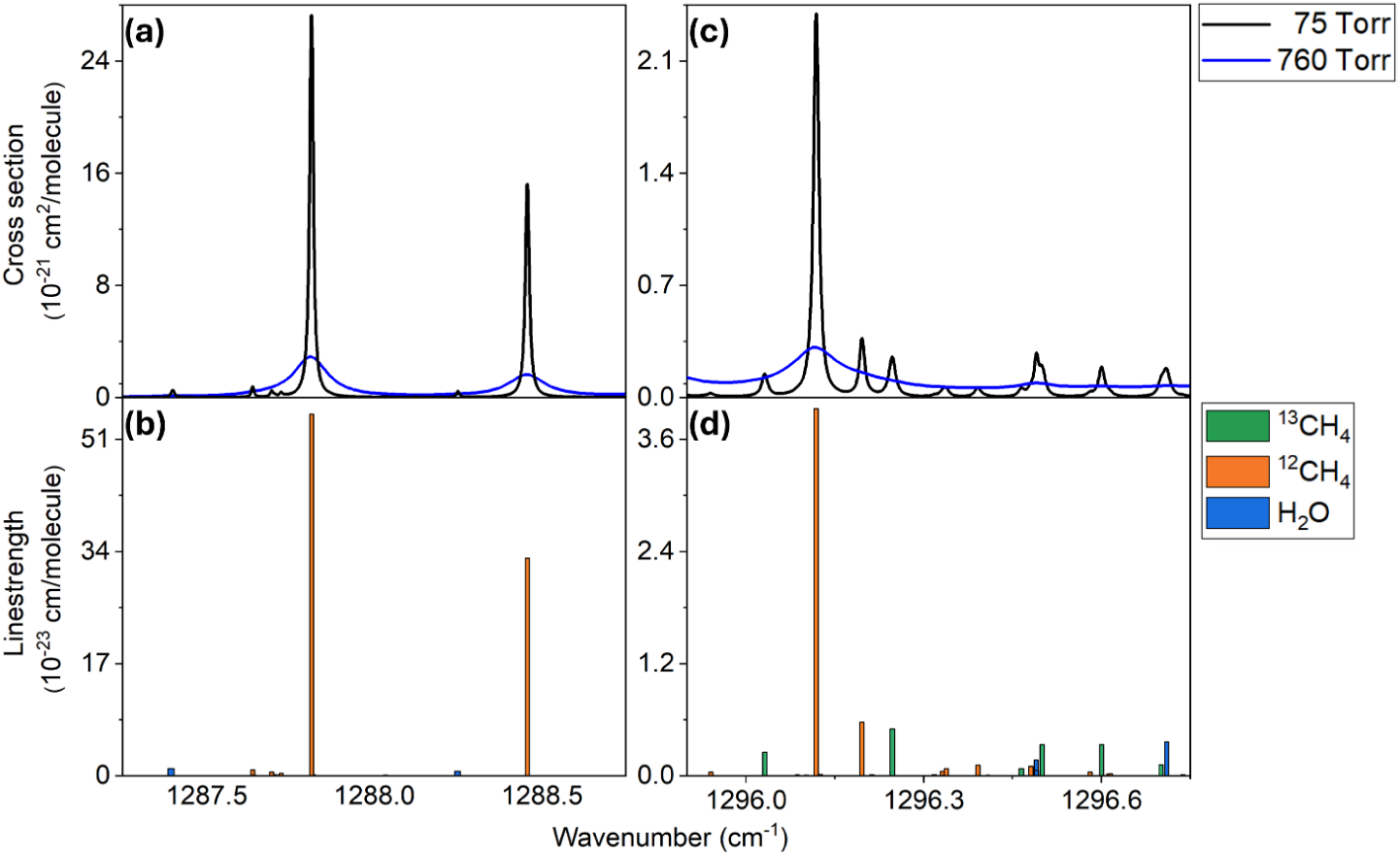
HITRAN-based simulation of (a) the absorption cross-section of the mixture over QCL1’s tuning range at 75 and 760 Torr, (b) the HITRAN database is used to simulate the line strength (on the y-axis) of the absorption lines of each separate analyte, multiplied by the analyte concentration in the mixture, over QCL1’s tuning range, (c) the absorption cross-section of the mixture over QCL2’s tuning range at 75 and 760 Torr, and (d) the line strength of the absorption lines of each separate analyte, multiplied by the analyte concentration in the mixture, over QCL2’s tuning range.

Figure 2a shows the HITRAN-based simulation of the absorption spectra of the mixture over QCL1’s tuning range. Figure 2b depicts the line strength of the absorption lines of each separate analyte, multiplied by the analyte concentration in the mixture. This provides a one-to-one association between the mixture’s absorption features in Figure 2a and the corresponding molecular species. This spectral region is dominated by ^12^CH_4_ absorption. Hence, this laser was employed to target the ^12^CH_4_ transition located at 1288.45 cm^–1^, which has a line strength of 3.35 × 10^–22^ cm/molecule at the ^12^CH_4_ concentration of 0.989% and is free from interference with ^13^CH_4_ and H_2_O.

Panels c-d display the HITRAN-based simulation within the spectral range covered by QCL2, which is suitable for the selective detection of ^13^CH_4_ by targeting the absorption line at 1296.03 cm^–1^ (P1). The selected feature has a line strength of 2.52 × 10^–24^ cm/molecule at the ^13^CH_4_ concentration of 0.011% and does not interfere with H_2_O absorption lines. Moreover, simulation results indicate that lowering the pressure to 75 Torr enables complete spectral separation of this feature from the adjacent ^12^CH_4_ line at 1296.12 cm^–1^ (P2). In contrast, at atmospheric pressure, the two features completely merge. Once the absorption lines were selected, the characterization of the optimal working pressure was performed in order to maximize the ^13^CH_4_ P1 QEPAS signal while minimizing overlap between the P1 and P2 2f-QEPAS spectra. With this aim, QCL2 was tuned to excite the overtone mode of the spectrophone and target the spectral region from 1295.9 to 1296.3 cm^–1^. The QEPAS signal obtained within the pressure range of 75–225 Torr for a mixture containing 5% CH_4_ in N_2_ is shown in Figure 3. For each pressure value, both the modulation frequency and amplitude were optimized.

**Figure 3:**
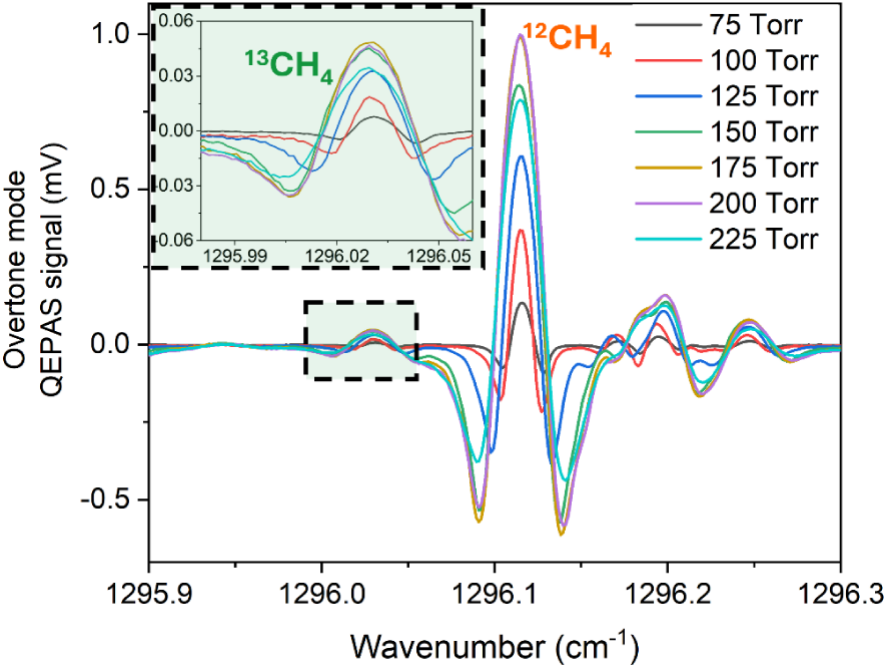
Acquired QEPAS signal in the spectral region from 1295.9 to 1296.3 cm^–1^, within the pressure range of 75–225 Torr for a mixture containing 5% CH_4_ in N_2_. These spectra were obtained by exciting the overtone mode of the spectrophone using QCL2.

The acquired spectral scans, normalized to the maximum detected peak signal, show the 2f signals corresponding to the ^13^CH_4_ transition P1 and the ^12^CH_4_ transition P2. The enlargement in the upper left corner highlights how the P1 peak increases with pressure up to 175 Torr, starting to decrease for higher pressure values. The QEPAS signal is proportional to the radiation to sound conversion efficiency, which increases with pressure, and to the quality factor, which decreases with pressure. The specific pressure at which a balance between these opposing effects occurs, which depends on the target molecule and the modulation frequency, was typically observed around 400 Torr for CH_4_ and a frequency of 17 kHz.^37,52^ Consequently, the decrease in the signal observed beyond 200 Torr is due to interference from the adjacent peak P2, which distorts the right negative lobe of P1 and reduces the overall peak signal. At both 75 and 100 Torr, P2 and P1 are fully resolved and spectrally separated. Although the peak signal P1 at 100 Torr is twice that at 75 Torr, the subsequent investigation was carried out at the lower pressure of 75 Torr. This choice ensures a lower background, as demonstrated in Figure 3.

The vibrational properties of the QTF coupled with the acoustic resonator tubes, namely, the resonance frequency and the quality factor of the two flexural modes, were measured by electrically exciting the QTF, both in pure N_2_ and in a matrix containing 5% CH_4_ in N_2_ at a pressure of 75 Torr. In both matrices, the fundamental mode exhibits a resonance frequency of *f*_0_= 2868.74 Hz and a quality factor of 13039, while the overtone resonance mode occurs at *f*_1_ = 17743.86 Hz with a quality factor of 28711.

### Sensor Calibration with Natural Abundance Methane Samples

First, the sensor was calibrated for CH_4_ detection using a cylinder of 10% of CH_4_ in N_2_ with certified isotopic composition matching CH_4_ natural abundance (9.89% of ^12^CH_4_ and 0.11% of ^13^CH_4_) and humidified N_2_ as the carrier gas. The sensor was operated in dual-gas mode to measure simultaneously the 2f spectra of both isotopologues. A small ramp around the selected ^12^CH_4_ peak at 1288.45 cm^–1^ and a sinusoidal modulation at *f*_0_/2 were applied to QCL1. Similarly, a small ramp around the ^13^CH_4_ selected peak at 1296.03 cm^–1^ with sinusoidal modulation at *f*_1_/2 was applied to QCL2. Figure 4a shows the QEPAS signal recorded by the *f*_0_ (upper panel, orange line) and *f*_1_ detection channel (lower panel, green line) for five different CH_4_ concentrations.

**Figure 4:**
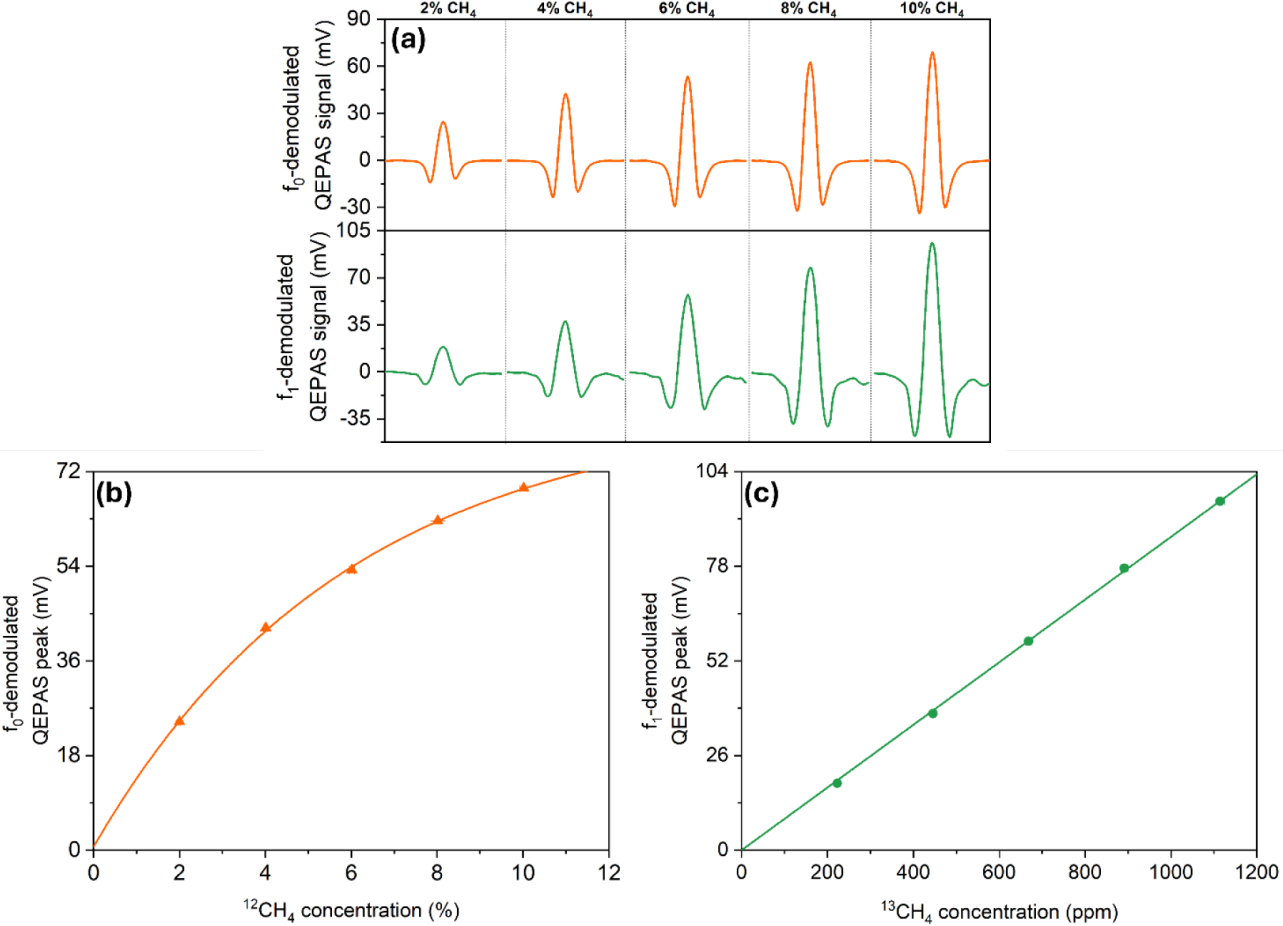
(a) Spectral scans of the selected ^12^CH_4_ absorption line at 1288.45 cm^–1^ (upper panel) and the ^13^CH_4_ absorption line at 1296.03 cm^–1^ (lower panel) for different CH_4_ concentrations in humidified N_2_. (b) *f*_0_-demodulated QEPAS peak vs ^12^CH_4_ concentration. Orange triangles: measured data. Solid line: fit with the function *y* = *Ae* ^*‑Bx*^ + *C*. (c) *f*_1_-demodulated QEPAS signal vs ^13^CH_4_ concentration, with QTF excited at the fundamental mode. Green circles: measured data. Solid line: linear fit.

The values of the *f*_0_- and *f*_1_-demodulated QEPAS signal at the two target absorption lines were averaged over five acquisitions and plotted against ^12^CH_4_ and ^13^CH_4_ concentrations, which were calculated by multiplying the overall CH_4_ concentration in the gas cylinder with the abundance of each isotopologue. The obtained results are listed in Figure 4b-c. The errors on the concentrations were calculated based on the extended uncertainty of the cylinder, reported as 1% of the nominal value. The *f*_0_-signal associated with the ^12^CH_4_ peak at 1288.45 cm^–1^ follows the Lambert–Beer law for nonweak absorptions in the % range, as shown in Figure 4b. The calibration curve for the sensor operating in fundamental mode was obtained by fitting the experimental data to an exponential function *y* = *Ae* ^–*Bx*^ + *C*, retrieving the best-fit parameters *A* = −83.42 ± 1.86 mV, *B* = 0.17 ± 0.02 1/%, and *C* = 84.06 ± 3.08 mV. Using these parameters, the *f*_0_-demodulated QEPAS signal can be expressed as a function of ^12^CH_4_ concentration, according to the following equation:

1 S0=−83.42mVe−0.171%×CH412+84.06mV

For CH_4_ concentrations above 4%, the *f*_1_-demodulated QEPAS signals associated with the ^13^CH_4_ peak (Figure 4a, lower panel) exhibit a small background on the right side of the spectra. This background, attributed to the stronger nearby P2 absorption line (not visible in the spectral scan), neither distorted the 2f scan nor affected the signal peak measurement. In fact, Figure 4c shows that the ^13^CH_4_ peak follows a linear trend against the ^13^CH_4_ concentration with an offset comparable to the noise level of the sensor. A linear fit was performed yielding a slope *m* = 0.0872 ± 0.0004 mV/ppm. Hence, the calibration curve for the sensor operating at the overtone mode is given by:

2 S1=0.0872mVppm×CH413

### Sensor Calibration with Nonstandard Isotopic Composition Methane Samples

The ultimate goal is to measure isotopic ratio deviations in various CH_4_ samples with nonstandard isotopic compositions. To achieve this aim, a series of measurements were conducted to evaluate the sensor response to varying ^13^CH_4_ concentrations in the presence of a fixed ^12^CH_4_ content.

The 10% CH_4_ and 280 ppm of ^13^CH_4_ cylinders were employed for this calibration, which consisted of two steps:

1. Measurement of the QEPAS signal recorded by the *f*_0_ and *f*_1_ detection channels for a mixture with a specific CH_4_ concentration and wet N_2_ for the rest.
2. Sequential addition of ^13^CH_4_ to the mixture, while maintaining a fixed CH_4_ concentration, with corresponding measurements of the QEPAS signals.

The ^12^CH_4_ concentration remained constant throughout the calibration at 0.989* CH_4_, based on the isotopic composition of the CH_4_ cylinder. The calibration procedure was repeated for four different total CH_4_ concentrations, CH_4_ = 0.2%, 1%, 2%, and 3%. For each concentration, the *f*_0_-demodulated ^12^CH_4_ QEPAS signal remains constant, with variations only within the noise level. This behavior further confirms that the sensor response at the fundamental mode depends exclusively on the ^12^CH_4_ concentration in the mixture. On the reverse, an increase in the *f*_1_-demodulated signal is observed as ^13^CH_4_ is added to the CH_4_ based mixture. For each CH_4_ concentration, the relationship between the *f*_1_-demodulated QEPAS peaks and the total ^13^CH_4_ concentration in the mixture was analyzed using the following equation:

3 $${{(}^{13}{\mathrm{CH}}_{4})}_{\mathrm{tot}}={\mathrm{CH}}_{4}\times 0.011+{{(}^{13}{\mathrm{CH}}_{4})}_{\mathrm{add}}$$

where the first term represents the concentration of ^13^CH_4_ naturally present in the CH_4_-based mixture and the second term represents the ^13^CH_4_ concentration added to the mixture. The obtained results are listed in Figure 5.

**Figure 5:**
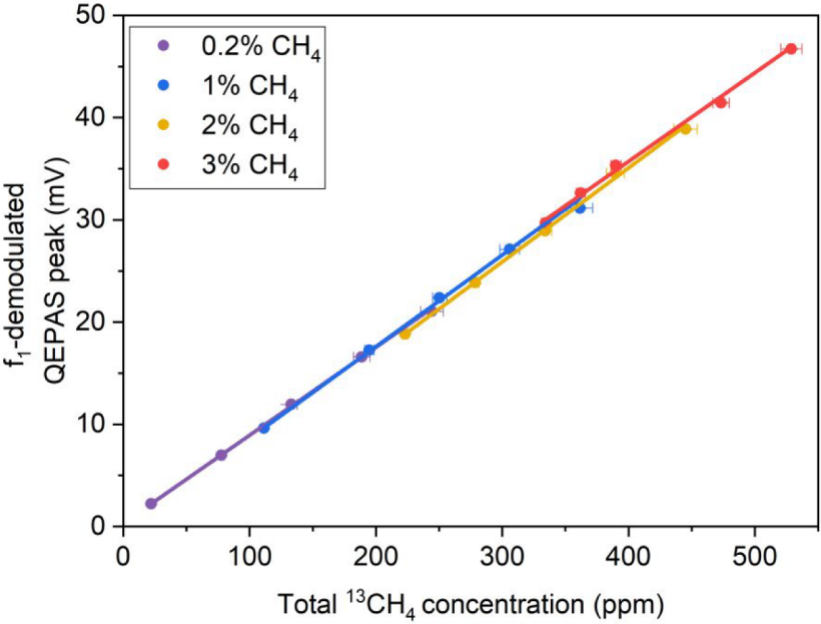
*f*_1_-demodulated QEPAS peaks vs total ^13^CH_4_ concentration. The concentration errors were calculated based on the extended uncertainties of the reference gas cylinders: 4% for the ^13^CH_4_ cylinder and 1% for the CH_4_ cylinder, both relative to their nominal values. Each curve represents a calibration obtained by fixing the CH_4_ concentration, indicated in the legend, and progressively adding ^13^CH_4_.

The first point in each series corresponds to the initial ^13^CH_4_ concentration before any ^13^CH_4_ addition ((^13^CH_4_)_add_ = 0). Subsequent points show the QEPAS signal related to progressive ^13^CH_4_ additions. A linear trend was verified for each calibration, each one corresponding to a different fixed concentration of CH_4_, and thus of ^12^CH_4_. The resulting slopes are comparable within the linear fit errors, with an average value of *m* = 0.0886 ± 0.0027 mV/ppm matching the slope of the CH_4_ calibration in N_2_ (Figure 4c) within the calculated uncertainty (see eq 2). These experimental results demonstrate that the *f*_1_-demodulated QEPAS signal depends exclusively on ^13^CH_4_ content in the mixture and remains independent of the ^12^CH_4_ concentration. This behavior provides evidence that nonradiative relaxation processes occur at a similar rate regardless of the presence of ^12^CH_4_ in the mixture. In mixtures containing only ^13^CH_4_ and humidified N_2_, energy relaxation proceeds through collisions with H_2_O and N_2_ molecules. When ^12^CH_4_ is also present, ^13^CH_4_ molecules can transfer their vibrational energy to ^12^CH_4_, which subsequently relaxes through the same collisional pathways.

### Sensor Performance in Measuring $\delta_{C}^{13}$

The above investigation clearly establishes that *f*_0_- and *f*_1_-demodulated QEPAS signals can be employed independently to retrieve the concentrations of ^12^CH_4_ and ^13^CH_4_ isotopologues, respectively, by inverting eqs 1 and 2. Hence, the dual-gas-sensing architecture can be employed for the complete characterization of the isotopic composition of unknown CH_4_ samples. For this application, the most appropriate configuration to retrieve the instantaneous isotope ratio consists in the on-peak detection applied to both *f*_0_ and *f*_1_ demodulation channels.

As a proof of concept, the sensor response to samples containing a fixed ^12^CH_4_ and a variable ^13^CH_4_ concentration was investigated. With this aim, the injection currents of QCL1 and QCL2 were set to match the ^12^CH_4_ and ^13^CH_4_ absorption peaks at 1288.45 and 1296.03 cm^–1^, respectively. While the CH_4_ concentration was fixed at 1% (0.011% of ^13^CH_4_ and 0.989% of ^12^CH_4_), ^13^CH_4_ was incrementally added to the mixture to generate five methane-based samples with variable isotopic ratios. During the measurements, the *f*_0_- and *f*_1_-demodulated QEPAS signals were continuously recorded and subsequently converted into ^12^CH_4_ and ^13^CH_4_ concentration values using eqs 1 and 2. The obtained results are listed in Figure 6.

**Figure 6:**
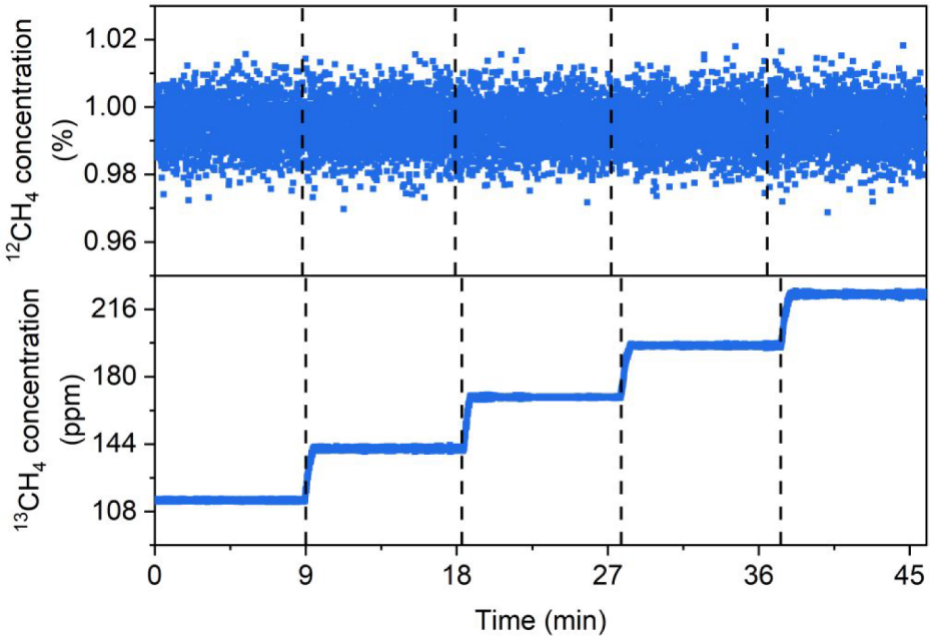
^12^CH_4_ and ^13^CH_4_ concentrations recorded by the dual-gas QEPAS sensor for five different methane-based samples. The samples were obtained by fixing the CH_4_ concentration at 1% (0.011% of ^13^CH_4_ and 0.989% of ^12^CH_4_) and incrementally adding ^13^CH_4_ to the mixture.

In Table 1, the concentrations retrieved from the QEPAS signals were compared to the expected values, which are reported in the table with their errors. The concentration errors on the experimental values were calculated based on the standard deviation of the recorded data (see Figure 6). The uncertainties in the expected concentrations were derived from the extended uncertainties of the reference gas cylinders: 4% for the ^13^CH_4_ cylinder and 1% for the CH_4_ cylinder, both relative to their nominal values.

**Table 1:** Comparison between the Concentrations Retrieved from the QEPAS Signals and the Expected Values, which Are Reported in the Table alongside Their Error, Calculated from Gas Cylinder Uncertainty

	**Sample**	**Measured**	**Expected**
**^12^CH_4_**			
	1–5	(0.994 ± 0.007) %	(0.989 ± 0.010) %
**^13^CH_4_**			
	1	(113.9 ± 0.4) ppm	(110 ± 1.1) ppm
	2	(141.4 ± 0.5) ppm	(138 ± 1.6) ppm
	3	(169 ± 0.3) ppm	(166 ± 2.5) ppm
	4	(196.5 ± 0.4) ppm	(194 ± 3.5) ppm
	5	(224 ± 0.5) ppm	(222 ± 4.6) ppm

The retrieved concentrations were in agreement with the nominal values within the experimental 3σ noise. This confirms the system’s reliability for quantifying isotopologue concentrations and evaluates isotopic ratios in unknown methane samples.

To assess the sensor capability in performing high-precision measurements of isotopic ratio deviation from CH_4_ standard samples, the sensor system sensitivity in detecting variations of the less abundant isotopologue ^13^CH_4_ was evaluated. The isotopic signature of a sample is characterized through the delta ratio $\delta_{C}^{13}$ , representing the deviation of the isotopic ratio of a sample with respect to the standard ratio (CH413CH412∼0.011) :

4 δC13=[(CH413CH412)sample(CH413CH412)standard−1]×1000‰

The sensitivity in estimating delta ratio variation, induced by fluctuations of the ^13^CH_4_ content while keeping the ^12^CH_4_ concentration constant, can be calculated through the error propagation:

5 ΔδC13=[(1CH412)sample(CH413CH412)standard]×ΔCH413×1000‰

The ^13^CH_4_ average noise equivalent concentration (Δ^13^CH_4_) can be retrieved from Table 1. By substituting this value in eq 5, we obtained a $\Delta \delta_{C}^{13}$ of approximately 3.5‰ for a 1% CH_4_ sample in N_2_ (^12^CH_4_ = 0.989%) at 100 ms integration time.

The essential requirement for providing valuable and reliable methane isotopic ratio deviations is the capability to sense ^13^CH_4_ variation with a precision of 1‰ or lower in a detection range as wide as possible. Since the sensitivity improves at longer integration times, a long-term stability analysis was conducted to experimentally assess the system’s performance in δ^13^C measurements. A 2 h long measurement (0.1 s lock-in integration time and 0.3 s acquisition time) was carried out in pure N_2_, at the working pressure of 75 Torr and with both laser currents fixed. The 2 h acquisition was then used to perform an Allan-Werle deviation analysis and evaluate the sensor noise at higher integration times. The retrieved values were converted in ${\Delta \delta }_{C}^{13}$ variations by employing eqs 2 and 5.

The obtained results are reported in Figure 7.

**Figure 7:**
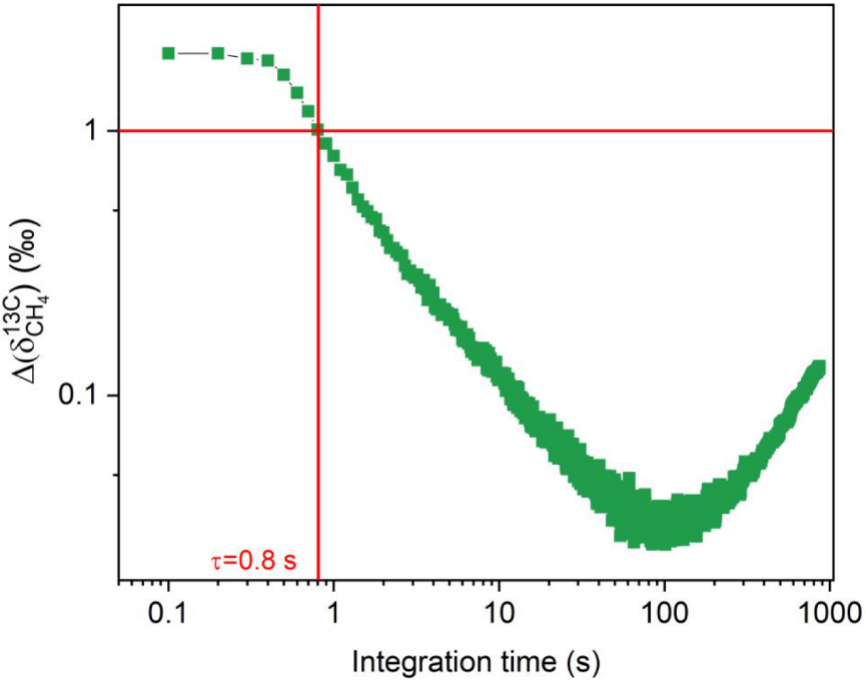
$\Delta \delta_{C}^{13}$ as a function of the integration time.

Figure 7 shows that the dual-gas QEPAS sensor achieves the 1‰ sensitivity standard at 0.8 s of integration time for a 1% CH_4_ content in a N_2_ matrix sample. These detection capabilities put the presented QEPAS spectrometer in the position to be competitive for isotopic measurements in natural gas characterization, while guaranteeing a sufficiently rapid response compatible with a real-time analysis. Moreover, these results can be extended to natural gas samples, provided that the effects of heavier hydrocarbons, such as ethane and propane, on methane’s QEPAS signal through energy relaxation interactions are properly accounted for.^40,53,54^

## Conclusions

The need to perform real-time, in-line measurements of isotopic ratios in gas mixtures requires the gas sample to be analyzed, with respect to the isotopologues of interest, within a small volume, preferably using a single sensing element, and with a response time compatible with the sampling flow rates. These requirements are fully met by the dual-gas QEPAS sensor demonstrated in the present study, which is dedicated to the measurement of δ^13^C in a methane sample diluted in nitrogen. This sensing system operates in the mid-infrared range and is capable of simultaneously detecting ^12^CH_4_ and ^13^CH_4_. The sensor exploits a frequency-modulated multiplexing scheme, employing two DFB QCLs to excite the fundamental and overtone modes of a QTF coupled with resonator tubes. Specifically, the excitation of the QTF at its fundamental vibrational resonance (*f*_0_) is employed for detecting ^12^CH_4_ while the overtone mode targets ^13^CH_4_ (*f*_1_). Sensor calibration using CH_4_ at natural isotopic abundance and ^13^CH_4_ enabled the characterization of the dependence of the *f*_0_- and *f*_1_-demodulated QEPAS signals on the respective isotopologue concentrations. This dual-channel detection strategy allows for simultaneous and interference-free monitoring of both methane isotopologues, enabling a comprehensive characterization of the isotopic composition in unknown methane samples. The sensor prototype proved a precision of 1‰ in measuring delta ratio variations induced by changes in ^13^CH_4_ concentration for a 1% CH_4_ sample at a 0.8 s integration time. These results are very promising for potential applications of the QEPAS multiplexing scheme in characterizing the isotopic composition of methane samples and monitoring processes involving isotopic fractionations. Moreover, the flexibility of the system allows for its extension to other isotopologues of interest and more complex sample matrices, including natural gas, where multiple hydrocarbon species and varying physical conditions may influence the signal response. To address these challenges, advanced compensation strategies can be employed, which include continuous monitoring of different analyte concentrations and physical parameters during the measurements, with a subsequent implementation of complex algorithms or multivariate approaches. By integrating such analytical techniques, the robustness, accuracy, and field applicability of the QEPAS sensor platform can be significantly enhanced, paving the way for its deployment in a wide range of scientific and industrial scenarios requiring precise isotopic methane sensing.

## ABBREVIATIONS

PAS: photoacoustic spectroscopy
QEPAS: quartz-enhanced photoacoustic spectroscopy
QTF: quartz tuning fork
QCL: quantum cascade laser
TDLAS: tunable diode laser absorption spectroscopy
FTIR: Fourier Transform Infrared
GC-MS: gas chromatography–mass spectrometry
GC-IRMS: gas chromatography–isotope ratio mass spectrometry
V-T: vibration-to-translation
WMDM: wavelength-modulation division multiplexing
ARs: acoustic resonators
ADM: acoustic detection module
CD/TEC: current driver/thermoelectric cooler
WFG: waveform generator
DAQ: data acquisition card
fwhm: full width at half-maximum
